# Application of tip-bendable ureteral access sheath in flexible ureteroscopic lithotripsy: an initial experience of 224 cases

**DOI:** 10.1186/s12894-023-01347-x

**Published:** 2023-11-01

**Authors:** Hui Liang, Lijian Liang, Yiwei Lin, Yin Yu, Xiaoling Xu, Zihao Liang, Jinmin Sheng, Baihua Shen

**Affiliations:** 1 Department of Urology, Xinchang County Hospital of Traditional Chinese Medicine, Shaoxing, Zhejiang Province China; 2 Department of Urology, the 1st Affiliated Hospital, School of Medicine, Zhejiang University, Hangzhou, Zhejiang Province China

**Keywords:** Lithotripsy, Ureteroscopy, Ureteral access sheath

## Abstract

**Introduction:**

During the last decades, the advent of flexible ureteroscopic lithotripsy has revolutionized the management of upper urinary tract stones. We designed a patented tip-bendable ureteral access sheath to facilitate stone clearance. Our current study reported our initial experience of 224 cases.

**Materials and methods:**

The study is a descriptive, retrospective analysis. The initial 224 cases, operated consecutively by one surgeon during 16 months, were reviewed. The novel tip-bendable ureteral access sheath was applied in the procedure. Demographics, laboratory tests, and peri- and postoperative findings (operation duration, stone-free rate (SFR), utilization of flexible instruments and complications) were analyzed.

**Resutls:**

The median age of the patients was 56 years and the mean stones size was 2.3 ± 1.3 cm. There were 63 cases of upper ureteral stone, 93cases of renal stone and 68 cases of ureteral-renal stones. The mean operative time was 69.2 ± 65.2 min. The immediate stone-free rate was 76.8% and the 1 month post-operative stone-free rate was 97.3%. Most cases(95.5%)were success in single session. Two patient experienced post-operative fever. There was no unplanned readmission. The frequency of post-operative complications was estimated at 0.89% (Clavien I).

**Conclusion:**

Flexible ureteroscopic lithotripsy with tip-bendable ureteral access sheath is a safe and effective procedure, which can achieve excellent stone clearance.

## Introduction

The urinary tract calculi are an important health problem with an incidence of 1–20% worldwide [1]. During the last decades, urologists are searching for the most efficient and minimally invasive surgical procedure for stone disintegration. Meanwhile, developments in endourological technologies make the retrograde intrarenal surgery (RIRS) more appealing for the majority of renal stones. It is recommended by the European Association of Urology (EAU) as the first recommended choice for renal stones ≤ 2 cm [2]. With the innovation in endourology, RIRS has been applied in some centers for more complex stones. Takazawa et al. [3] reported 100% stone free rate (SFR) handling RIRS for 2-4 cm renal stones. Riley et al. [4] showed a 90.9% success rate for stones averaging 3 cm in size. Naoya et al. [5] applied RIRS as a primary treatment for staghorn calculi, which could accomplish a final 48% SFR for patients with > 60 mm calculi.

However, the limited SFR, the necessity of multiple surgical sessions and the potentially life-threatening complications related to intra-renal pressure remain the major restrictions for such procedure [6], especially while managing larger kidney calculus. Intending to overcome the shortages of RIRS, we designed a patented ureter access sheath with features of suction and bendable-tip. The tip of the innovated ureteral access sheath (UAS) can reach the target calyx along with the flexible ureteroscope. Thus, it can facilitate dusts and fragments flushing. Applying the sheath extensively enhances the stone clearance while maintaining the intrarenal pressure. Herein, we summarized our initial experience of 224 cases as below.

## Materials and methods

The medical files of 224 patients who were treated for ureteral or renal calculi during Oct 2021 and Nov 2022 in Xinchang County Hospital of Traditional Chinese Medicine and 1st Affiliated Hospital of Zhejiang University, School of Medicine, Zhejiang Province, China were retrospectively reviewed. All the patients were diagnosed with upper urinary tract calculi by preoperative imaging studies including CT scan. Patients with a congenital renal anomaly, previous urological surgery and refractory infection were excluded. Stone size and location were evaluated preoperatively by non-contrast CT scan. Stone size was measured in its largest diameter. When multiple stones presented, the sum of diameters was recorded as stone burden. Preoperative laboratory tests included routine CBC, urine analysis, urine culture, serum creatinine test, coagulation panel and basic metabolic panel including blood glucose test.

All procedures were performed under general or continuous epidural anesthesia by one single expert surgeon. Patients with positive urine cultures were treated with appropriate antibiotics preoperatively until thecultures turned to be negative. For others, broad-spectrum antibiotics were applied peri-operatively for prophylaxis. Most patients scheduled for RIRS were pre-stented 7–14 days prior to the RIRS surgery in outpatient surgery.

RIRS was performed in the dorsal lithotomy position. After D-J stent retrieval, a 0.035-inch guidewire was introduced into the upper urinary tract. Ureteroscopic inspection was performed. If ureter stone presented, it was fragmented and flushed back to the renal pelvis. A 12/14F tip-bendable UAS (Elephant II, Zhejiang YiGao Medical Technology Co. Ltd, Hangzhou, China) was inserted over the guidewire. A 9.9 F digital flexible ureteroscope (URF-V, Olympus) was advanced along with the UAS into the renal pelvis or the targeted calyces. For the cases with stones in the lower calyx, basket was applied for stone relocation when the infundibulopelvic angle (IPA) was steep. Stones were fragmented with 200 μm holmium laser fibers (Raykeen Laser Technology Limited Corporation, Shanghai, China) under specific energy setting of 1-1.5 J and a rate of 15–20 Hz.

All patients were evaluated on postoperative days 1 by CT to assess stone-free status. For those failed to gain stone-free status, an additional CT was required on postoperative day 30 as further evaluation. Stone-free was defined as the absence of any stones or residual fragments ≤ 2 mm under non-contrast CT [5]. The operative time was counted from the beginning of lithotripsy to the end of the surgery. Complications were evaluated to the Clavien classification.

All statistical analyses were performed using a standard software package (Stata, version. 11.0, StataCorp). Descriptive analysis was performed to evaluate distribution patterns of patients’ demographics, stone characteristics, and operation data. Operation time was recorded as mean ± standard deviation (SD) and examined using a Kruskai-Wallis *H* Test. Categorical variables were expressed as percentages. Chi-square or Fisher’s exact Test was used to compare the pairs of categorical variables. The analysis considered significant when *P* < 0.05.

## Results

Demographics and preoperative data are shown in Table 1. Overall, ages of the patients ranged from 19 to 68 years old with a median age of 56 years old. There were 93 patients with renal calculi only and 63 cases with ureteral calculi only. The other 68 cases were diagnosed with concomitant renal and ureteral calculi. In the patients with renal calculi, there were 96 cases with lower calyx involved. Most patients were pre-stented to ensure the accurate placement of UAS. There were 10 patients required planned multiple surgical sessions because of massive stone burden. For these patients with multiple surgical sessions, the operative time for each session were amounted. The mean operative time was 69.2 ± 65.2 min. Immediate stone clearance was achieved in 172 (76.8%) cases. The SFR on postoperative day 30 was 97.3%. The overall complication rate in the study group was extremely lower. Post-operative fever (Clavien Ia) occurred in 2 patient and it was successfully managed by potent antibiotics.

**Table 1:** Peri-operative data of 224 cases

Peri-operative parameters	Value
Stone size (cm)	2.2 ± 1.3
Hounsfield Unit Value	1019 ± 328
Stone Distribution	
Renal Stone Only	93
Ureteral Stone Only	63
Renal and Ureteral Stone	68
Lower calyx involved	96
Pre-stented	220
Operation time(min)	69.2 ± 65.2
Use of basket(%)	32.6
Complications	
Clavien grade I	2
Clavien grade II-IV	0
Stone-free rates	
Immediate	76.8%
30 days postoperative	97.3%

The data was further stratified by stone burden, stone location and Hounsfield unit values (Table 2). We identified that SFR dramatically dropped when the stone size increased. For the patients with stone size ≤ 3 cm, the surgical duration and SFR was excellent. However, in the subgroup with stone size > 3 cm, the operation time prolonged (157.5 ± 108.1 min) and the final SFR decreased to 84.2%. Ten cases (26.3%) even required additional surgical session. The localization of stone did not significantly affect the final SFR. However, cases with lower calyx involvement consume more operation time. More baskets were applied in these cases. Totally, basket was utilized in 73 (32.6%) cases in our case series for stone relocation prior to laser lithotripsy and fragments extraction. In the subgroup with lower calyx stone, half cases needed basket for assistance. The Hounsfield unit values failed to present any significant effect on SFR. However, stone with Hounsfield unit > 1200 consumed more surgical time and would need additional surgical session more frequently.

**Table 2:** Stratified peri-operative data

	n	Operation time(min)	Immediate SFR	SFR 30 days postoperative	multiple sessions	No. of basket(%)
Stone size						
<2 cm	125	40.1 ± 22.4	84.8%	100.0%	0	31(24.8)^*^
2-3 cm	61	73.7 ± 28.9^*^	77.0%	100.0%	0	28(45.9)
>3 cm	38	157.5 ± 108.1^*^	50.0%^*^	84.2%^*^	10^*^	14(36.8)
Lower calyx involved						
No	90	48.0 ± 42.7	88.9%	97.8%	2	6(6.7)
Yes	134	83.3 ± 73.5^*^	68.6%^*^	97.0%	8	67(50.0) ^*^
Hounsfield Unit Value						
≤1200Hu	146	58.9 ± 49.8	80.8%	98.6%	3	48(32.9)
>1200Hu	78	88.3 ± 84.0^*^	69.2%	96.1%	7^*^	25(32.0)

*P < 0.05

## Discussion

Recently, the improvement in flexible endoscopes, the accessories, and laser technology have made RIRS one of the most popular surgical choices in the management of upper urinary calculi [7, 8]. Thulium fiber laser (TFL), disposable flexible ureteroscope and suctioning UAS are considered as 3 game changers for RIRS [7]. Unfortunately, RIRS is restricted by several drawbacks. First, unlike PCNL, RIRS can not clear the fragments immediately. The requirement of multiple surgical sessions and unexpected re-admission are the main concerns. Even more, RIRS might cause the formation of steinstrasse while managing larger stone, which may require a series of surgical interventions [9]. The removal of small, asympatomatic kidney stones during surgery resulted in fewer subsequent emergency department visits, surgeries and stone regrowth in randomized controlled trial [10]. Second, the prolonged operation time and the rising intrarenal pressure would lead to reflux, and subsequently increase the risk of hemorrhage and infection [11]. One of the most important innovations to overcome these drawbacks is the application of UAS [7]. UAS allows multiple entrance of ureteroscope and facilitates active removal of the stone fragments during the procedure. However, with conventional UAS, the back-flow mainly depends on scope-sheath ratio [12] and the baskets should be used for fragments retrieval frequently.

To improve initial SFR of RIRS, several strategies have been introduced to optimize the procedure. Bryniarski et al. [13] introduced the method by modifying the position of the patient to relocate lower pole stones. Multescu et al. [14] recommended that fragmented the stone to the extractable fragments was the optimal lithotripsy method for stones larger than 1 cm because the dust may hinder visualization of the clear operative field and the difficulty of differentiating a small fragmented stone in the midst of dust. Currently, the vacuum assisted UAS greatly improved the efficiency. It is able to aspirate the tiny fragments during lithotripsy simultaneously. Zeng et al. [15] revealed that suction UAS could improve stone clearance, optimize visual field and reduce stone retropulsion. Chen et al. [16] introduced a novel method to aspirate the fragments directly by suction UAS combined with artificial saline circulation, which could reduce the use of baskets and thus decrease the operation time and medical costs.

**Fig. 1:**
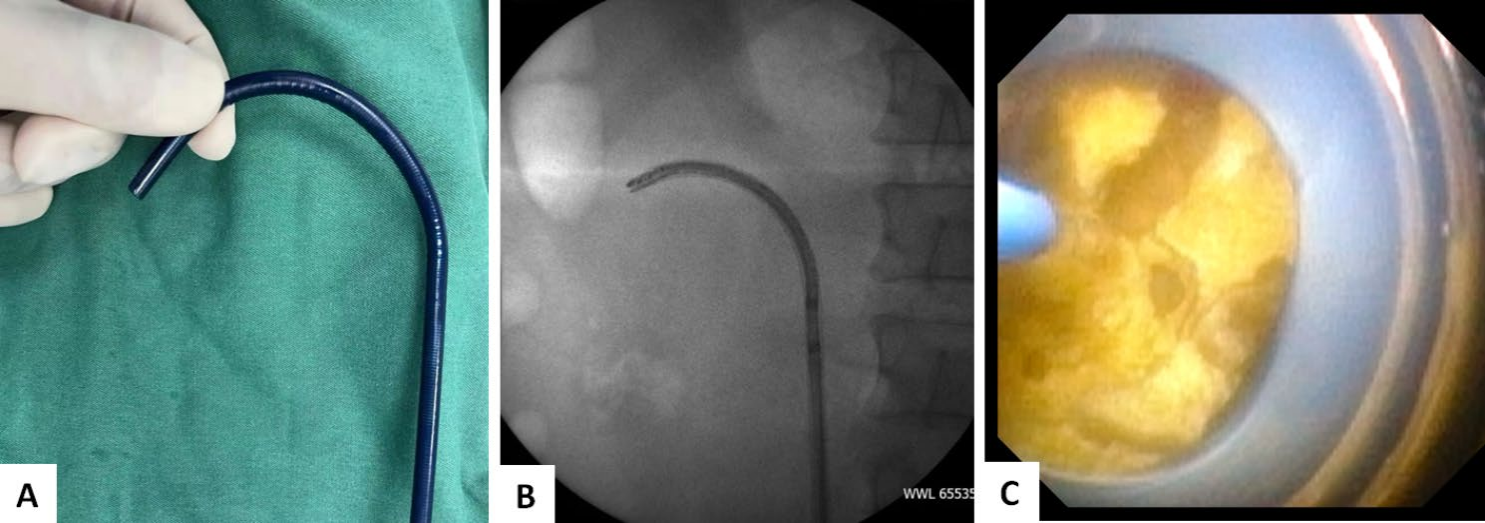
The proximal 10 cm tip of the novel UAS is bendable **(A)**. The UAS can be passively deflected and navigated into the targeted calyx **(B)** or the stone surface **(C)**

**Fig. 2:**
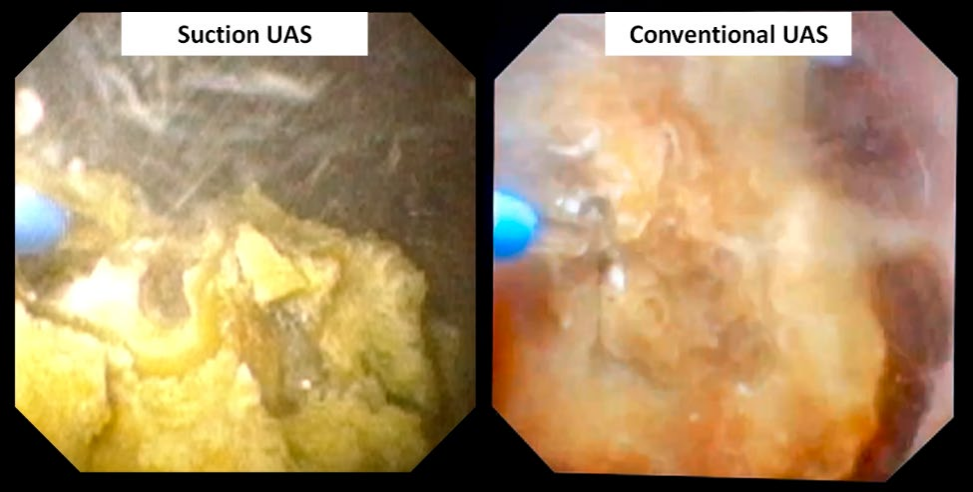
The tip-bendable suction UAS can provide better surgical vision while comparing with the conventional UAS

However, the conventional suction UAS usually cannot pass through the ureteropelvic junction (UPJ) and get close to the stone. So the aspiration effect will be weakened and the efficiency of fragments clearance is limited. The application of our tip-bendable UAS can overcome the shortcoming from the conventional suction UAS. The proximal 10 cm tip of our novel UAS is bendable. It can cross over the UPJ and be passively navigated to the targeted renal calyx or stone surface (Fig. 1). The oblique suction-evacuation channel can provide continuous aspiration effect towards specific calyx or fragments, thus made the fragments being washed out by the vortex flow much more efficiently while maintaining a clear operative vision (Fig. 2). It can efficiently remove fragments, which can achieve an ideal stone free rate of 97.3% in our case series. The tip-bendable UAS is compatible to regular vacuum system. It is easy to be manipulated and the learning curve is steeper.

In our current case series, we also identified the following features. First, stone size does matter a lot. Benefiting from the direct suction effect of tip-bendable UAS, the potential for SFR can approach those of percutaneous surgery for small to moderate-sized stone. In our current study, for patients with stone size ≤ 3 cm, both surgical duration and SFR were comparable with PCNL as reported in the literatures [17, 18]. However, the enlarged stone size deteriorates the SFR and consumes more operative time. For larger stone (> 3 cm), the final SRF is dramatically reduced to 84.2% and operative time is significantly prolonged. Larger stone will also bring extra surgical session for the patients. However, since it can maintain low intra-renal pressure during the whole procedure, which made the RIRS a safe approach for stone with larger size. In all of our cases, only two patients suffered from fever.

Second, the maximal deflection of the tip-bendable UAS is limit and depends on flexibility of ureteroscope. It cannot reach the inferior calyx when the IPA is sharp. In such cases, accessing the lower pole to treat the stone in situ becomes difficult. So, when inferior calyx stone presented, baskets should be applied for stone relocation or fragments retrieval. In the subgroup with lower calyx stone, the basket was used much more frequently.

Third, the effect of stone density was also assessed. Hounsfield density used to be an important parameter to predict the outcome of RIRS [19]. In our study, we assigned 1200 Hu as a specific threshold to stratify the hardness into two categories. However, there were no significant differences in operative time, SRF and basket usage between the two categories. Possibly, because our laser setting is effective enough to handle all types of stones. As we have noticed, for stone with Hounsfield density > 1200 Hu, more fragments would be produced. We should withdrawal the scope much more frequently to facilitate fragments aspiration during the procedure, which pose the scope to the risk of damage and lower the efficiency. Two strategies may further optimize the procedure. Laser with efficient dusting property, such as TFL, will generate smaller particles and obtain higher stone clearance [20, 21]. Smaller caliber endoscopes will bring an ideal scope-sheath ratio allows larger fragments to be aspirated out without scope withdraw, thus enhance the stone clearance [22].

Our current study also had several limitations. The study was in retrospective observational design. Since it was our initial clinical experience, the sample size was still small. So, our study was insufficient to validate the superiority of current technique over conventional techniques. Larger-scale prospective comparative study should be conducted to confirm our hypothesis. Besides, most cases in our study were pre-stented to facilitate the UAS insertion. Smaller caliber UAS cooperated with novel endoscopes may overcome the drawback.

## Conclusion

Our present study showed that RIRS with tip-bendable UAS was promising in the management of upper urinary tract stones. It is safe and effective. Prospective randomized trials would be required to further delineate the superiority over conventional RIRS and PCNL.

## Data Availability

The original data in the current study was available from the corresponding author on reasonable request.
